# Vector mapping and bloodmeal metabarcoding demonstrate risk of urban Chagas disease transmission in Caracas, Venezuela

**DOI:** 10.1371/journal.pntd.0010613

**Published:** 2023-03-17

**Authors:** Maikell Segovia, Philipp Schwabl, Salem Sueto, Candy Cherine Nakad, Juan Carlos Londoño, Marlenes Rodriguez, Manuel Paiva, Martin Stephen Llewellyn, Hernán José Carrasco

**Affiliations:** 1 Instituto de Medicina Tropical, Universidad Central de Venezuela, Caracas, Venezuela; 2 School of Biodiversity, One Health Veterinary Medicine, University of Glasgow, Glasgow, United Kingdom; 3 Escuela de Salud Pública, Universidad Central de Venezuela, Caracas, Venezuela; Federal University of Ceará, Fortaleza, Brazil, BRAZIL

## Abstract

Chagas disease is a significant public health risk in rural and semi-rural areas of Venezuela. Triatomine infection by the aetiological agent *Trypanosoma cruzi* is also observed in the Metropolitan District of Caracas (MDC), where foodborne *T*. *cruzi* outbreaks occasionally occur but active vector-to-human transmission (infection during triatomine bloodmeal) is considered absent. Citizen science-based domiciliary triatomine collection carried out between 2007 and 2013 in the MDC has advanced understanding of urban *T*. *cruzi* prevalence patterns and represents an important public awareness-building tool. The present study reports on the extension of this triatomine collection program from 2014 to 2019 and uses mitochondrial metabarcoding to assess feeding behavior in a subset of specimens. The combined, thirteen-year dataset (n = 4872) shows a high rate of *T*. *cruzi* infection (75.2%) and a predominance of *Panstrongylus geniculatus* (99.01%) among triatomines collected in domiciliary areas by MDC inhabitants. Collection also involved nymphal stages of *P*. *geniculatus* in 18 of 32 MDC parishes. Other collected species included *Triatoma nigromaculata*, *Triatoma maculata*, *Rhodnius prolixus*, and *Panstrongylus rufotuberculatus*. Liquid intestinal content indicative of bloodmeal was observed in 53.4% of analyzed specimens. Dissection pools representing 108 such visually blooded *P*. *geniculatus* specimens predominantly tested positive for human cytochrome b DNA (22 of 24 pools). Additional bloodmeal sources detected via metabarcoding analysis included key sylvatic *T*. *cruzi* reservoirs (opossum and armadillo), rodents, and various other synanthropic and domesticated animals. Results suggest a porous sylvatic-domiciliary transmission interface and ongoing adaptation of *P*. *geniculatus* to the urban ecotope. Although *P*. *geniculatus* defecation traits greatly limit the possibility of active *T*. *cruzi* transmission for any individual biting event, the cumulation of this low risk across a vast metropolitan population warrants further investigation. Efforts to prevent triatomine contact with human food sources also clearly require greater attention to protect Venezuela’s capital from Chagas disease.

## Author summary

Vector-borne diseases are a significant cause of morbidity and mortality in tropical and subtropical countries. Efficient control strategies require detailed understanding of vector species distribution and behavior in human environments. General public awareness-building that selected animal species carry and transmit dangerous human-infective parasites is also key. This study details long-term spatiotemporal prevalence patterns of hematophagous Chagas disease vectors known as triatomines based on the collection of specimens from domiciliary areas of greater Caracas, the capital of Venezuela. A citizen science-based sample collection approach is used to simultaneously help educate the public on Chagas disease and prevention. Microscopy indicates that triatomines found inside homes by inhabitants of the city are often positive for the Chagas disease parasite *Trypanosoma cruzi*. Specimens found inside homes also occasionally involve nymphs. Such evidence of indoor reproduction is found in various parts of the city. Genetic analyses of triatomine intestinal content further indicate that domiciliary triatomine occurrence is facilitated by feeding on human blood. Bloodmeals on various other domesticated and wild animal species are also identified, suggesting an extensive *T*. *cruzi* transmission network spanning urban and sylvatic environments. Results compel greater attention toward Chagas disease surveillance in Caracas. A human serological survey for *T*. *cruzi*, especially in the pediatric population, would help clarify the consequences of triatomine infection and human-triatomine contact events demonstrated from this study’s citizen science-based dataset.

## Introduction

Chagas disease, also known as American trypanosomiasis, is a life-threatening vector-borne zoonosis caused by *Trypanosoma cruzi*, a protozoan parasite endemic to various regions of the tropical and subtropical Americas [1]. Transmission occurs when infected hematophagous triatomine insects (Reduviidae, subfamily Triatominae [2,3]) defecate during bloodmeal, releasing metacyclic parasite stages into the bite wound or intact mucosal membranes. Apart from this active (stercorarian) mode of transmission, human infection can result congenitally (mother to fetus), via blood transfusion from an infected donor, or upon ingestion of food (often fruit juices) contaminated with triatomines or their feces [1]. Predation is also considered an important alternative to stercorarian forms of *T*. *cruzi* transmission among a vast range of non-human hosts. The parasite has been recorded in at least 73 mammalian genera occurring in diverse sylvatic, peri-domiciliary, and domiciliary ecotopes [4].

Venezuela currently belongs to the countries most heavily burdened by Chagas disease. Recent estimates of *T*. *cruzi* seroprevalence exceed 10% nationwide, with highest values suggested for hotspots of active transmission in Barinas, Lara, Portuguesa, and Trujillo states [5,6]. Although case burden is primarily concentrated to the country’s rural Andean piedmont, the possibility that human *T*. *cruzi* infections are also being contracted in larger urban centers deserves greater attention. Urban infection events become possible when cities encroach onto wild *T*. *cruzi* habitat without effective barriers against human-vector contact and when environmental conditions allow vectors not only to temporarily survive in domiciliary settings but also to colonize these long-term without needing to return to the wild for food and reproduction. Regular urban circulation of *T*. *cruzi* may then begin to establish if urban triatomine food sources represent *T*. *cruzi*-competent host species or when competent host species commonly enter the urban space from the wild.

The above conditions appear relevant to the Metropolitan District of Caracas (MDC), Venezuela’s capital region of several million inhabitants and little to no regular *T*. *cruzi* sero-surveillance in recent decades [6,7]. The MDC features some of the largest shantytowns in Latin America (both along its perimeter and stretching into the city center) and borders directly on expansive sylvatic environment such as Waraira Repano National Park. Multiple foodborne outbreaks have occurred in and around the MDC in the past and have recurrently involved *Panstrongylus geniculatus*, one of 24 triatomine species found across Venezuela [8–11]. Despite its extensive geographic distribution (mid-Central America to northern Argentina), *P*. *geniculatus* has historically been considered a predominantly sylvatic species of low epidemiological importance in the Americas relative to species presenting high levels of (peri-) domiciliation in multiple populations [12]. These include *Rhodnius prolixus* and *Triatoma dimidiata* in northern South America and Central America, *Triatoma infestans* and *Panstrongylus megistus* in the Southern Cone, and *Triatoma brasiliensis* in Brazil [12]. Possibly influenced by intervention campaigns targeting these species and other processes of climate and environmental change [13], changes in ecological niche and dispersal behaviors are increasingly reported for *P*. *geniculatus*, also including evidence of domiciliary adaptation in parts of Venezuela and Brazil [14–17]. Molecular evidence of human blood feeding by *P*. *geniculatus* in the MDC and the adjacent states of Miranda and La Guaira (formerly called Vargas) was first shown in 2005, as intestinal contents from 53 of 88 analyzed specimens reacted positively to human antiserum. Of these, 41% were positive for *T*. *cruzi* infection [18]. *Panstrongylus geniculatus* is considered a poor *T*. *cruzi* vector on account of its slow digestion (ca. 1 hour between start of feeding and excretion in adults) [19], but the assumption that active Chagas disease transmission is therefore absent from the MDC may need reconsideration if human blood feeding by *T*. *cruzi*-infected triatomines recurrently affects many MDC residents.

To improve general understanding of urban triatomine ecology and help measure the public health risk posed by species such as *P*. *geniculatus* in the MDC, the Instituto de Medicina Tropical (IMT) at Universidad Central de Venezuela (UCV) has operated a citizen science-based triatomine collection program since the year 2000. MDC inhabitants that observe triatomines in domiciliary areas are encouraged to submit specimens to the program for taxonomic identification and *T*. *cruzi* infection analysis [20]. Safe collection protocols and various other educational materials are provided at http://www.chipo.chagas.ucv.ve. The first program report describes results for the collection years 2007 to 2013 [21]. Of 3551 triatomines analyzed for this time period, 75.2% were infected with *T*. *cruzi* and nearly all (98.96%) identified as *P*. *geniculatus*. Nymphal triatomine stages, a strong indicator of local reproduction, were also found inside homes in 15 of the MDC’s 32 parishes [21].

The present work represents an update on results from the IMT triatomine collection program, now covering data spanning 2007 to 2019. This update also describes triatomine biting reports by MDC inhabitants and uses cytochrome b (cytb) metabarcoding to provide a snapshot of triatomine blood feeding patterns in the parishes with highest triatomine collection yield. Bloodmeal metabarcoding (most often targeting cytb and 12S) is widely used in the study of Chagas disease vector ecology [22–30]. For example, this tool has helped infer triatomine dispersal rates between wild and human-inhabited ecotopes (e.g., cases of high connectivity and re-infestation potential shown for *T*. *infestans* in Bolivia [22] and *T*. *dimidiata* in Ecuador [23]), or gauge triatomine diet specialization and overlap (e.g., within [24] and across triatomine species in Colombia [25]).

Results of this study suggest that *P*. *geniculatus* has continuously occurred in domiciliary spaces throughout most of the MDC over a surveillance period of 13 years. Metabarcoding analyses highlight humans as a primary blood source among domiciliary specimens collected via the citizen science approach. The domiciliary sample set shows exceptionally high rates of *T*. *cruzi* infection, possibly facilitated by contact with various reservoir species (e.g., *Didelphis marsupialis*, *Dasypus novemcinctus*, and *Rattus* spp.) also detected via bloodmeal analysis. Results call for greater attention to human infection rates in the MDC, where *T*. *cruzi* seroprevalence has not been directly assessed in recent years. Although acute Chagas disease can be highly symptomatic, it is possible that autochthonous cases are occurring without report and treatment given limited access to medical care. We hope that this study stimulates greater attention towards the study and management of Chagas disease and numerous other public health burdens that Venezuela has been facing in the past decade.

## Materials and methods

### Triatomine collection

The present study summarizes previously published triatomine collection metrics representing the period 01/01/2007–12/31/2013 [21] in conjunction with results of new collection analyses representing the period 01/01/2014–12/31/2019. The study was performed under approval of the IMT ethical committee and the National Council of Scientific and Technological Research of Venezuela. All triatomines were brought to the IMT by MDC inhabitants as part of the citizen science-based collection program introduced above. Information recorded from contributors included name, address, and phone number for all collection years. Beginning in 2014, contributors were also asked whether the specimen was collected following a suspected biting event or collected independently of any such interaction. Suspected bites were reviewed by medical staff. The MDC is a political administrative division composed of 5 municipalities and 32 parishes covering an area of 810 km^2^ at an average of 900 meters altitude with a population of 2.95 million inhabitants according to the 2011 census [7] (present day population size has not been officially recorded but may differ significantly due to extensive population movement in and out of Venezuela over the past decade).

### Triatomine identification and parasite screening

Specimens collected between 2007 and 2019 were identified to species and sex using the morphological key by Lent and Wygodzinsky [11]. Stomach and intestines were drawn into isotonic saline solution (0.85%) on microscope slides using watchmaker’s forceps. Samples were then stained with Giemsa and examined microscopically for the presence of parasites. Characteristic morphological forms of *T*. *cruzi* were identified as described by Hoare [4]. The presence or absence of liquid content within the intestines (potential bloodmeal) was also noted during the dissection process.

### Bloodmeal barcoding sample set origin

Mitochondrial DNA metabarcoding was used to profile the bloodmeal content of 108 adult *P*. *geniculatus* specimens (40 male and 68 female) obtained from citizen science contributions to the IMT between 2009 and 2016 (S1 Table). The sample represents a random subset of geo-referenced specimens presenting blood-like intestinal content from Petare (n = 47), Baruta (n = 29), and Sucre (n = 32). Sample size reflects study budget. Petare, Baruta, and Sucre parishes differ socioeconomically and in the extent and quality of urban development (Instituto Nacional de Estadística (INE) [7]). Petare on the northeastern side of the MDC contains one of the largest, most densely populated shantytowns in Latin America (ca. 11200 inhabitants per km^2^ (INE)). Much of Petare is characterized by uncontrolled building practices with limited (green) space between units. Baruta in the southern MDC features higher levels of civil planning in most urban areas, with significantly lower population densities (ca. 3600 inhabitants per km^2^ (INE)). Baruta more often features larger property units separated from others by areas of green space. Urban aggregations are also more often interrupted by extensive areas of sparsely occupied, naturally forested land. Sucre occupies the northwestern corner of the MDC and is likewise less densely populated (ca. 6700 inhabitants per km^2^ (INE)) and more environmentally heterogeneous than Petare, extending from its high-density core (Catia neighborhood) into sparsely populated montane areas on the southern edge of Waraira Repano National Park. Urban aggregations also occur along subsections of La Guaira highway leading northwest out of Sucre to the international airport [31]. Aside from these broad characterizations from the literature, this study did not attempt to define environmental differences between parishes given substantial heterogeneity within parishes.

### Cytochrome b amplicon library construction

Total DNA extracted from triatomine digestive tracts [32] (Promega Wizard Genomic Purification Kit, USA) was brought to equimolar concentrations and consolidated into 24 pools according to *T*. *cruzi* infection status, collection period (2009–2012 vs. 2013–2016), parish, and sex (S1 Table).

A conserved, ~359 bp region of the cytb gene [33] was amplified by polymerase chain reaction (PCR) using forward primer 5’-ACACTGACGACATGGTTCTACACCATCCAACATCTCAGCATGATGAAA-3’ and reverse primer 5’-TACGGTAGCAGAGACTTGGTCTGCCCCTCAGAATGATATTTGTCCTCA-3’. This first-round PCR reaction creates 22 nt overhangs required for subsequent barcoding PCR using universal forward primer 5’-AATGATACGGCGACCACCGAGATCTACACTGACGACATGGTTCTA-3’ and barcoded (*X*) reverse primer (5’-CAAGCAGAAGACGGCATACGAGAT*X*TACGGTAGCAGAGACTTGGTCT-3’. Two DNA extraction controls (dH_2_O) were also subjected to cytb- and barcoding PCR. The final library was purified using AMPure XP paramagnetic beads (Beckman Coulter).

### Cytochrome b amplicon sequence analysis

The 24 amplicon pools and 2 negative controls underwent paired-end 2 x 300 nt sequencing on the Illumina MiSeq platform using Reagent Kit v3, yielding between 159907 and 476984 read-pairs for all pools except pool 12 (9188 read-pairs) and 17 (60926 read-pairs). After quality-filtering by 5’-3’ windowed trimming (-q 25) with sickle v1.33 [34], forward and reverse reads were merged and clustered to consensus haplotypes with the ‘cluster_otus’ command (-otu_radius_pct 3) in USEARCH v8.1 [35]. Potential cross-talk signal was mitigated by discarding consensus haplotypes from a pool if the consensus haplotype was supported by less than 200 reads within the pool but by more reads in other pools. Consensus haplotypes were then defined to species by searching for ≥90% matches to cytb sequences databased by the National Center for Biotechnology Information at https://www.ncbi.nlm.nih.gov/nucleotide/. The 2 negative controls introduced during DNA extraction showed no signal following the procedures described above.

Apart from the 97% similarity threshold-based clustering methods used for between-species classification, we also resolved unique haplotypes from human DNA signal found in the dataset using DADA2 v1.2.2 [36] in R v3.4.1. The DADA2 algorithm infers a parametric model of substitution errors from the data and then determines whether variation around abundant sequence types is statistically consistent or inconsistent with the expected error distribution. Inconsistent reads form new clusters that represent distinct sources of DNA. We applied standard filtering parameters (truncQ = 2, maxEE = c(2, 2)) and performed 3’-trimming using truncLen = c(230, 230).

The positions of mutations distinguishing human haplotypes found using DADA2 were manually identified via sequence alignment and consistency to known human mitochondrial diversity reviewed at https://www.mitomap.org [37].

Finally, we also assessed the relationship between human sample size and number of segregating sites in cytb target alignment by randomly subsampling Latin American mitogenomes sequenced by the 1000 Genomes Project [38] accessible at ftp.1000genomes.ebi.ac.uk. We downloaded multi-fasta files representing 3 available Latin American foci: Lima, Peru; Medellín, Colombia; and Puerto Rico (all municipalities). The dataset from Puerto Rico might constitute the best available proxy for human cytb target diversity in Caracas given similar mitochondrial haplotype distribution observed in prior studies [39,40]. Greater mitochondrial divergence is possible for Lima and Medellín due to limited historic gene flow between populations of the Caribbean region and those of the Andes and Pacific coast [40]. We used custom scripts to draw 100 random cytb target segments (excluding primer binding sites) for every possible sample size from each multi-fasta file. Segregating sites were extracted after each draw using SNP-sites v2.1.3 [41] and average counts plotted with standard deviations using base graphics in R v3.4.1.

## Results

### Geographical distribution of triatomines in Caracas

General collection results are browsable in a dynamic dashboard provided in S1 File.

**Fig 1:**
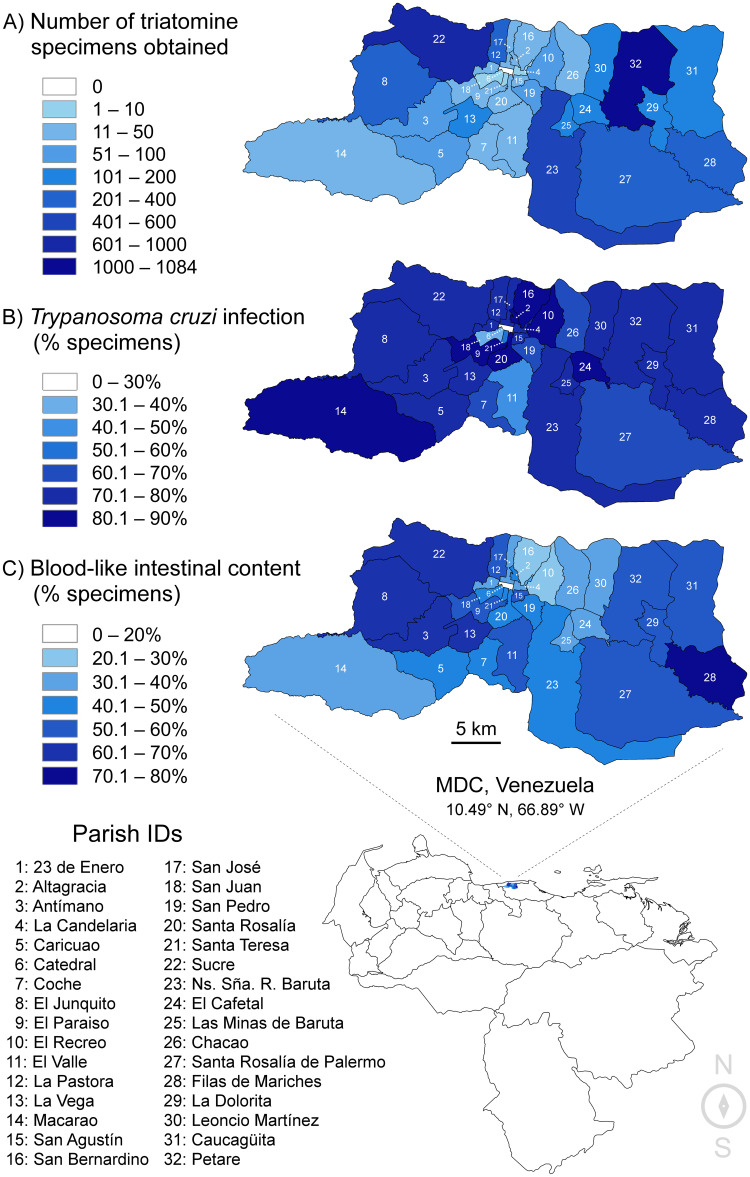
**A. Numbers of triatomine specimens obtained from each MDC parish for the collection period 2007–2019.** Fill color in the choropleth map represents the total number of specimens obtained per parish, increasing from lighter to darker blues. No specimens were obtained from Catedral parish (#6, white fill). A total of 4872 specimens were obtained across 31 parishes. **B. Rates of natural *T*. *cruzi* infection in triatomine specimens from each MDC parish for the collection period 2007–2019.** Fill color in the choropleth map represents the rate of *T*. *cruzi* infection (by microscopy) per parish, increasing from lighter to darker blues. Infection occurred in all parishes from which triatomine specimens were obtained. The analyzed sample set represents 3845 specimens. **C. Rates of observing liquid material (potential bloodmeal) in triatomine intestinal content from each MDC parish for the collection period 2007–2019.** Fill color in the choropleth map represents visually blooded triatomine occurrence per parish, increasing from lighter to darker blues. The visually blooded condition occurred in all parishes from which triatomine specimens were obtained. The analyzed sample set represents 4422 specimens. A random subset of visually blooded specimens from Petare (#32), Baruta (#23), and Sucre (#22) feature in bloodmeal barcoding analysis. The country map at bottom was created using 1:10 m cultural vectors from https://www.naturalearthdata.com. MDC maps above contain information from OpenStreetMap and OpenStreetMap Foundation, which is made available under the Open Database License. Specifically, parish boundaries were plotted using administrative level 7 lines downloaded with the OpenStreetMap Overpass API [42].

**Fig 2:**
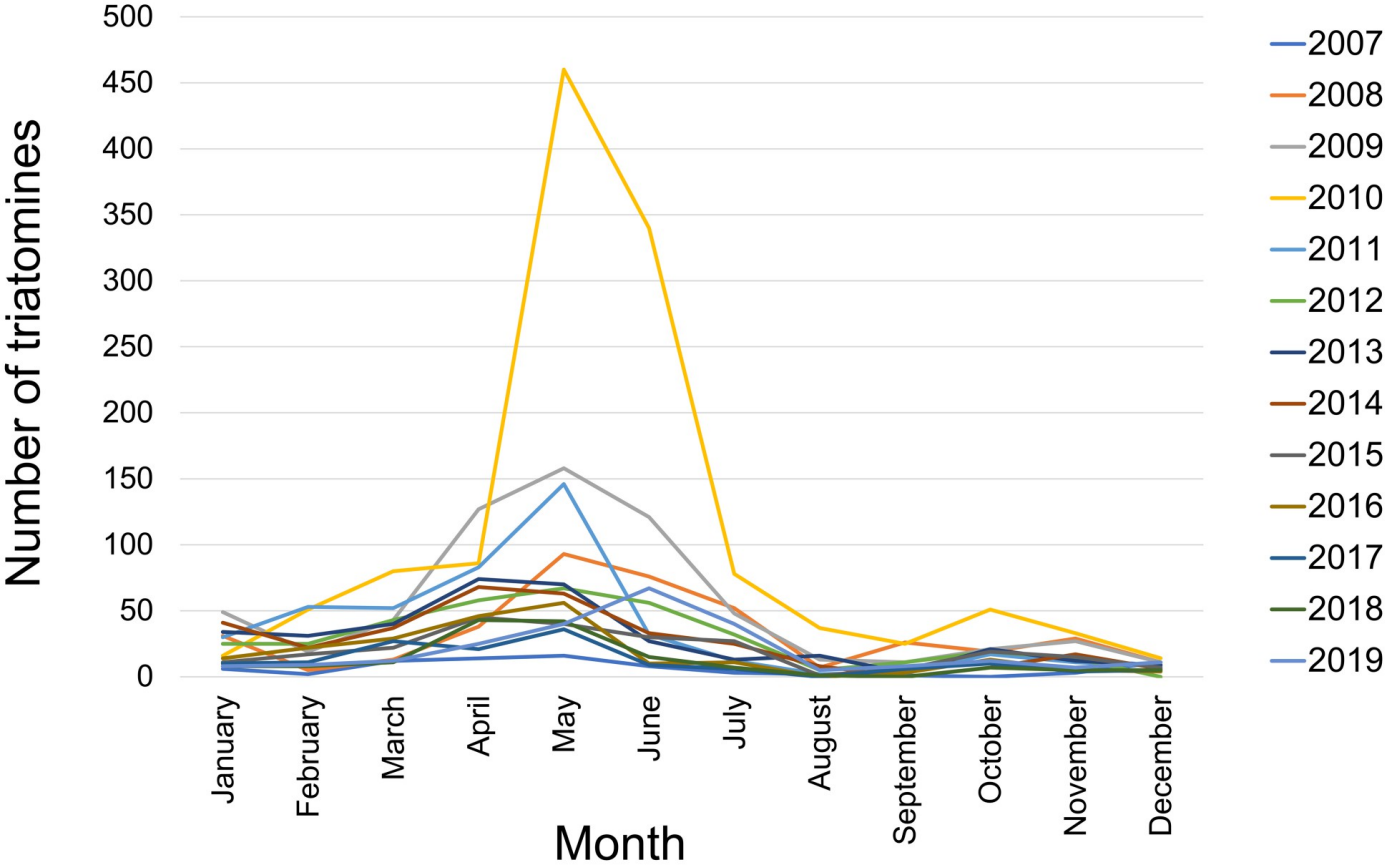
Monthly triatomine yield for the MDC collection period 2007–2019. Line color indicates collection year. Media attention to an oral Chagas disease outbreak in May 2010 may help explain the high collection numbers of May and June 2010.

A total of 1321 triatomines were analyzed for the period 2014–2019. Of these, 1310 (99.17%) were taxonomically identified as *P*. *geniculatus*, 3 (0.23%) as *Triatoma maculata*, 5 (0.38%) as *Triatoma nigromaculata*, 1 (0.08%) as *R*. *prolixus*, and 2 (0.15%) as *Panstrongylus rufotuberculatus*. These metrics ​​are highly similar to those demonstrated previously for the period 2007–2013. Species composition for the combined, 2007–2019 dataset (4872 triatomines) was 4824 (99.01%) *P*. *geniculatus*, 17 (0.35%) *T*. *maculata*, 26 (0.53%) *T*. *nigromaculata*, 3 (0.06%) *R*. *prolixus*, and 2 (0.04%) *P*. *rufotuberculatus*. The geographical distribution of triatomines collected between 2007 and 2019 spans 31 of 32 MDC parishes. Only Catedral parish yielded no specimens during the study period. The highest numbers of specimens were obtained from Petare, Sucre, and Baruta parishes (Fig 1A and S1 File and Table 1). Fig 2 shows the monthly record of triatomines for the period 2007–2019, the time of highest collection rates occurring between April and July.

**Table 1:** Numbers of triatomine specimens obtained from each MDC parish for the collection period 2007–2019, tabulated by species. Slashes separate values for adult (left of slash) and nymphal triatomine specimens (right of slash). Hyphens (-) are used when no specimens of the indicated species were obtained from the parish. The IDs used for parishes correspond to those used in Fig 1.

ID	Parish	*Panstrongylus geniculatus*	*Triatoma maculata*	*Triatoma nigromaculata*	*Rhodnius prolixus*	*Panstrongylus rufotuberculatus*	Total
1	23 de Enero	15/1	-	-	-	-	**16**
2	Altagracia	38/1	-	-	-	-	**39**
3	Antímano	91/7	-	-	1/0	-	**99**
4	La Candelaria	5/0	-	-	-	-	**5**
5	Caricuao	83/2	1/0	-	-	-	**86**
6	Catedral	-	-	-	-	-	**0**
7	Coche	19/0	1/0	-	-	-	**20**
8	El Junquito	286/5	2/0	4/0	-	-	**297**
9	El Paraiso	36/0	-	-	-	-	**36**
10	El Recreo	52/0	-	-	-	-	**52**
11	El Valle	22/0	-	-	-	-	**22**
12	La Pastora	311/8	-	-	-	-	**319**
13	La Vega	115/4	2/0	-	-	-	**121**
14	Macarao	25/0	-	-	-	-	**25**
15	San Agustín	15/0	-	-	-	-	**15**
16	San Bernardino	29/0	-	-	-	-	**29**
17	San José	50/0	-	-	-	-	**50**
18	San Juan	4/0	-	-	1/0	-	**5**
19	San Pedro	50/0	1/0	-	-	-	**51**
20	Santa Rosalía	11/0	-	-	-	-	**11**
21	Santa Teresa	7/0	-	-	-	-	**7**
22	Sucre	578/40	1/0	-	-	-	**619**
23	Ns. Sña. R. Baruta	526/9	2/0	4/0	-	-	**541**
24	El Cafetal	171/3	-	-	-	-	**174**
25	Las Minas de Baruta	96/8	1/0	-	-	-	**105**
26	Chacao	35/2	0/1	-	1/0	-	**39**
27	Sta. Rosalía de Palermo	307/9	3/0	9/0	-	1/0	**329**
28	Filas de Mariches	254/14	2/0	2/0	-	-	**272**
29	La Dolorita	98/7	-	-	-	-	**105**
30	Leoncio Martínez	185/5	-	-	-	-	**190**
31	Caucagüita	104/2	-	3/0		-	**109**
32	Petare	1033/46	-	4/0	-	1/0	**1084**
	Total	4651/173	16/1	26/0	3/0	2/0	**4872**

### *T*. *cruzi* infection rates in triatomines

**Fig 3:**
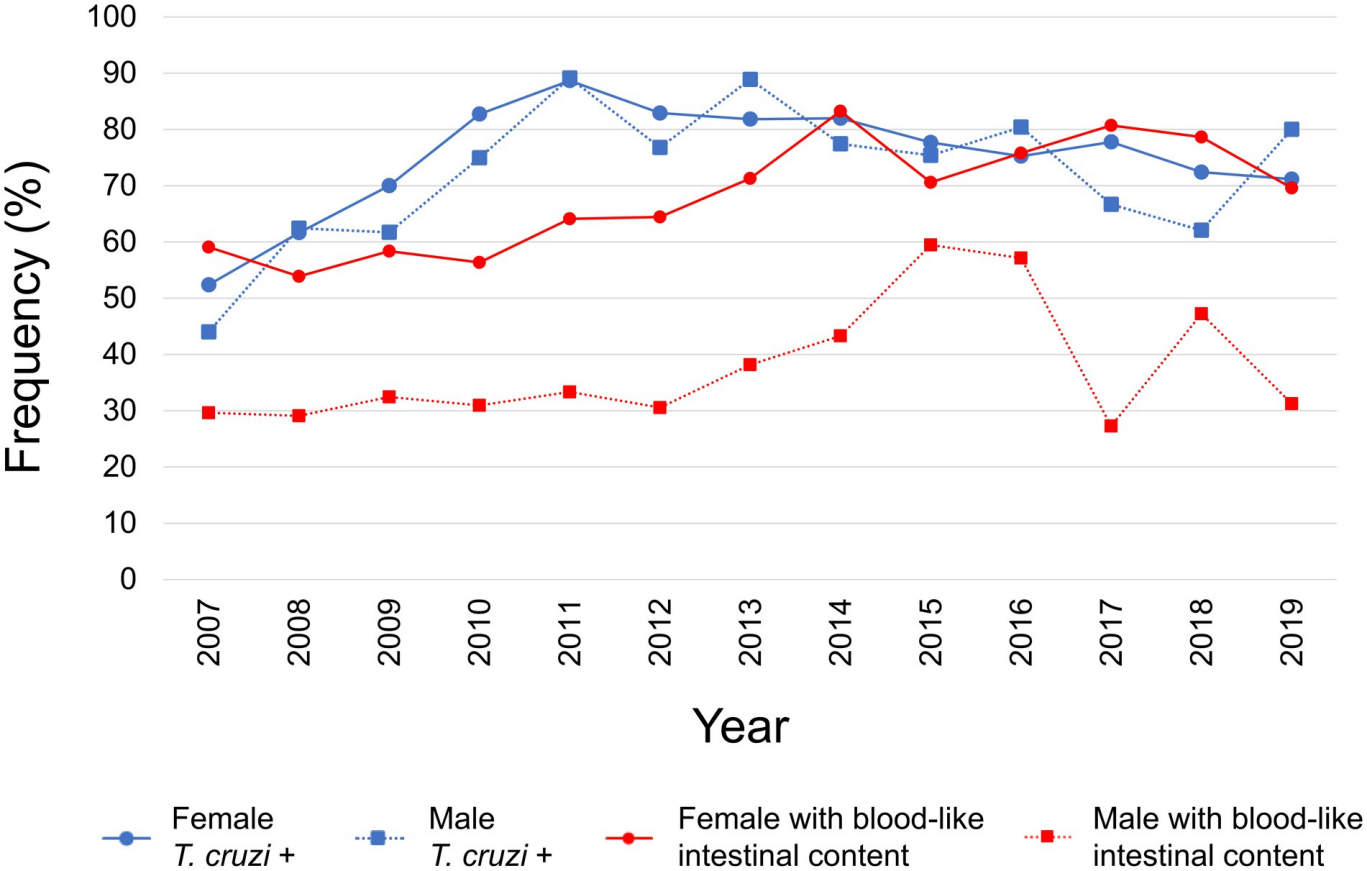
Annual rates of natural *T*. *cruzi* infection and observations of blood-like intestinal content in male and female triatomine specimens for the MDC collection period 2007–2019. Blue color indicates the rate of *T*. *cruzi* infection among 3845 specimens analyzed. Red color indicates the rate at which blood-like intestinal content was observed among 4422 specimens analyzed. Circles and squares indicate values for females and males, respectively.

Of 4872 studied triatomines, 3845 (78.9%) could be evaluated for natural infection with *T*. *cruzi*. The remaining insects were either in a state of internal decomposition or too dry at the time of microscopic analysis. These 3845 specimens included 3711 adults, of which 55.8% (2146) were females and 40.7% (1565) were males. Rates of natural infection by *T*. *cruzi* were 77.2% (1657/2146) for females and 73.9% (1156/1565) for males. These rates are categorized by year in Fig 3 and S2 Table. They are categorized by parish in Fig 1B and S3 Table.

Infection by *T*. *cruzi* was 75.4% (2873/3810) for *P*. *geniculatus*, 47.4% (9/19) for *T*. *nigromaculata*, 50% (6/12) for *T*. *maculata*, 50% (1/2) for *R*. *prolixus*, and 100% (2/2) for *P*. *rufotuberculatus* (S2 Table).

### Presence of blood in triatomine digestive tracts

Presence or absence of liquid content in the intestines (potential bloodmeal) was determined for 4422 (90.8%) of 4872 triatomines. These 4422 specimens included 4270 adults, of which 2513 were females (58.9%) and 1757 were males (41.1%). Blood occurred in 65% (1633/2513) of these females and 34.5% (607/1757) of these males. The distribution of blood-containing triatomines is categorized by sex and year in Fig 3 and S4 Table, and by parish in Fig 1C and S5 Table. Only in the species *T*. *nigromaculata* did blood occur more often in the digestive tracts of males (5/7) than in those of females (7/15).

### Nymphal triatomine stages

Of the 4872 triatomines collected between 2007 and 2019, 174 (3.6%) were identified as nymphs (173 *P*. *geniculatus* and 1 *T*. *maculata*) (Table 1 and S1 File). These nymphs were found in 18 of the MDC’s 32 parishes, varying in number over the years. Follow-up questioning clarified that all nymphs were found inside houses, not in peripheral areas.

Of 134 nymphs received in adequate condition for the determination of infection with parasites, 78 (58.2%) were positive for *T*. *cruzi*. Rates of infection were 0% (0/3) for nymphal stage II (NII), 63.3% (19/30) for nymphal stage III (NIII), 60.3% (41/68) for nymphal stage 4 (NIV), and 54.5% (18/33) for nymphal stage V (NV) (S2 Table).

Liquid content within the intestines (potential bloodmeal) was detected in 123 (80.9%) of 152 evaluated nymphs. The presence of liquid content within the intestines was 100% (3/3) for NII, 80.6% (29/36) for NIII, 76.9% (60/78) for NIV, and 88.6% (31/35) for NV (S4 Table).

S6 Table shows the percentage of nymphs collected in relation to the total number of triatomines collected by parish per year. The parishes with the highest percentage of nymph collection across years were Chacao (7.7%), Minas de Baruta (7.6%), Antímano (7.1%), La Dolorita (6.7%), and Sucre (6.5%). The highest absolute numbers of collected nymphs across years were obtained from Petare (46), Sucre (40), and Filas de Mariche (14) parishes. It is important to emphasize that all nymphs were found within houses, most of them in the bedrooms.

### Blood feeding patterns

**Fig 4:**
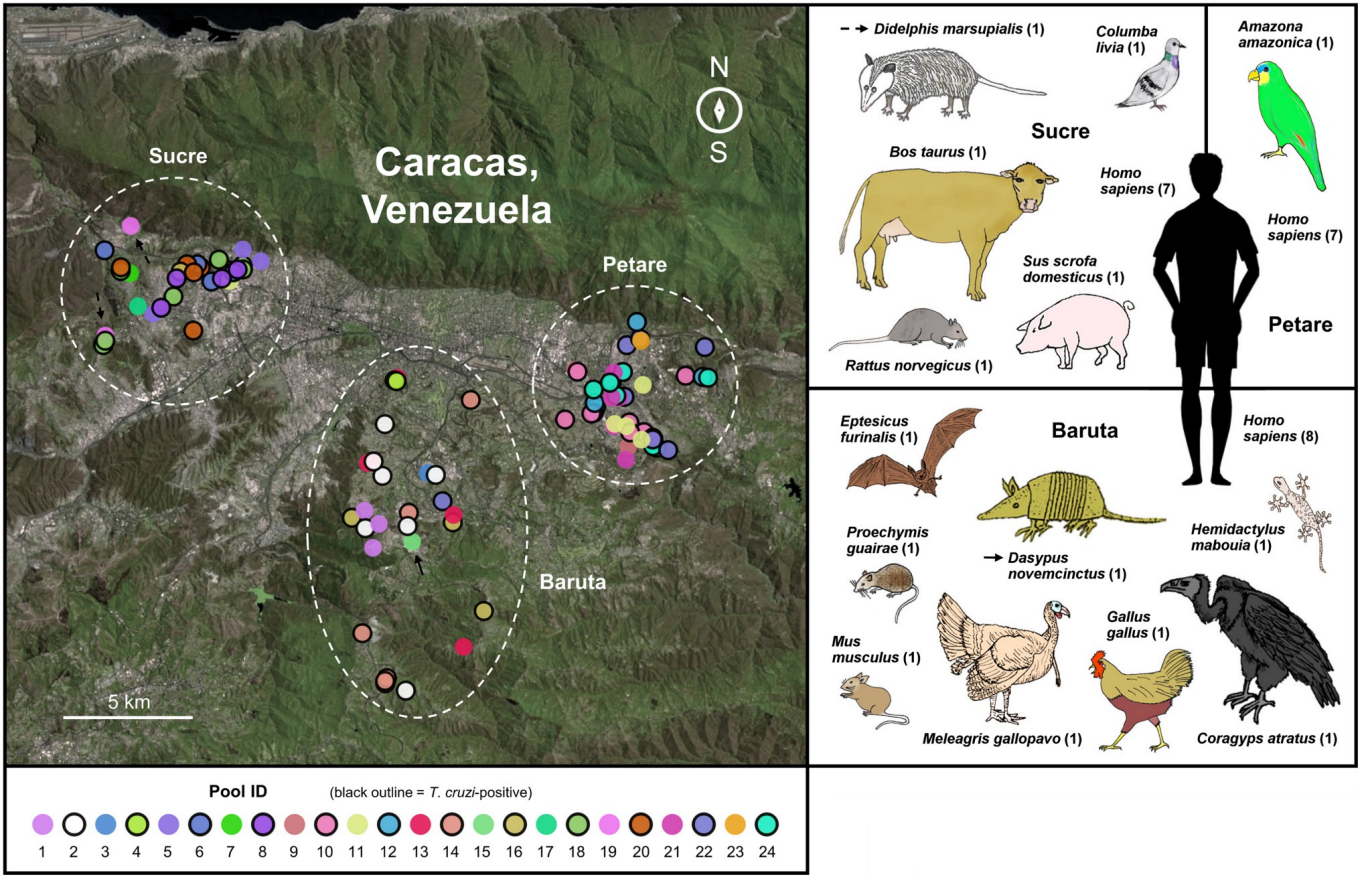
Vertebrate DNA sources identified in 108 pooled *P*. *geniculatus* bloodmeals from Petare, Baruta, and Sucre parishes for the MDC collection period 2009–2016. Points in the map indicate the geographical origin of the 108 samples contributing to the 24 dissection pools used in cytb analysis. Point fill color indicates pool membership. Pools composed of *T*. *cruzi*-positive samples are outlined in black. Arrows indicate pools presenting cytb DNA from the *T*. *cruzi* reservoir species *D*. *marsupialis* and *D*. *novemcinctus*. Numbers in parentheses indicate the number of pools in which the corresponding vertebrate blood source was detected. The map base layer represents Scene ID LC80040532015120LGN01 from the Landsat 8 OLI/TIRS C1 Level-1 dataset–courtesy of the U.S. Geological Survey.

Fifteen distinct bloodmeal sources were identified among the 24 sample pools (see Methods) representing 108 randomly selected, blood-containing adult *P*. *geniculatus* specimens collected between 2009 and 2016 (Tables 2 and 3, S1 Table and Fig 4 and S2 File). These included the major sylvatic *T*. *cruzi* reservoir species *Dasypus novemcinctus* (nine-banded armadillo) and *Didelphis marsupialis* (common opossum) (see arrows in Fig 4), 4 livestock/poultry species (*Sus scrofa domesticus*, *Bos taurus*, *Meleagris gallopavo*, *Gallus gallus*), 3 wild avian species (*Columba livia*, *Coragyps atratus*, *Amazona amazonica*), 3 rodent species (*Rattus norvegicus*, *Mus musculus*, *Proechimys guairae*), the brown bat *Eptesicus furinalis*, and the house gecko *Hemidactylus mabouia*.

**Table 2:** Vertebrate sources of cytb DNA identified in *P*. *geniculatus* bloodmeals for the MDC collection period 2009–2012. Dissection pools 1–12 are organized in columns based on the parish from which contributing samples were obtained. Pool sizes (i.e., the number of samples contributing to each pool) range from 1–7. Vertebrate species with cytb sequences matching pool sequences at ≥90% similarity are listed in rows numbered 1–4. Corresponding read support values are provided at right prior to the ‘Match’ column indicating the fraction of nucleotides matching between pool sequences and the reference (excluding primer binding sites).

Parish →	Baruta				Sucre				Petare				Match →
Pool size →	3	7	1	1	4	4	1	4	3	7	5	4	
Pool ID →	1	2	3	4	5	6	7	8	9	10	11	12	
1) *Homo sapiens*	1625	7122	512	8268	5153	5220	0	7205	5983	6015	7334	0	307/307
2) *Amazona amazonica*	0	0	0	0	0	0	0	0	0	0	2	0	302/307
3) *Mus musculus*	0	834	0	0	0	0	0	0	0	0	0	0	307/307
4) *Columba livia*	0	0	0	0	0	0	4075	0	0	0	0	0	307/307

**Table 3:** Vertebrate sources of cytb DNA identified in *P*. *geniculatus* bloodmeals for the MDC collection period 2013–2016. Dissection pools 13–24 are organized in columns based on the parish from which contributing samples were obtained. Pool sizes (i.e., the number of samples contributing to each pool) range from 1–12. Vertebrate species with cytb sequences matching pool sequences at ≥90% similarity are listed in rows numbered 1–12. Corresponding read support values are provided at right prior to the ‘Match’ column indicating the fraction of nucleotides matching between pool sequences and the reference (excluding primer binding sites).

Parish →	Baruta				Sucre				Petare				Match →
Pool size →	5	6	1	5	2	8	2	7	7	12	1	8	
Pool ID →	13	14	15	16	17	18	19	20	21	22	23	24	
1) *Homo sapiens*	6618	10100	8649	3309	355	10100	2426	8632	11900	9026	10100	9035	307/307
2) *Dasypus novemcinctus*	0	0	47	0	0	0	0	0	0	0	0	0	306/307
3) *Sus scrofa domesticus*	0	0	0	0	0	0	0	45	0	0	0	0	307/307
4) *Proechimys guairae*	21	0	0	0	0	0	0	0	0	0	0	0	304/307
5) *Eptesicus furinalis*	18	0	0	0	0	0	0	0	0	0	0	0	300/306
6) *Didelphis marsupialis*	0	0	0	0	0	0	15400	0	0	0	0	0	305/307
7) *Hemidactylus mabouia*	0	0	0	20	0	0	0	0	0	0	0	0	307/307
8) *Meleagris gallopavo*	17	0	0	0	0	0	0	0	0	0	0	0	306/307
9) *Bos taurus*	0	0	0	0	0	0	0	5	0	0	0	0	307/307
10) *Coragyps atratus*	7	0	0	0	0	0	0	0	0	0	0	0	304/306
11) *Rattus norvegicus*	0	0	0	0	0	642	0	0	0	0	0	0	307/307
12) *Gallus gallus*	0	0	517	0	0	0	0	0	0	0	0	0	307/307

While the above-listed species were detected in only 1 pool each and predominantly in Baruta parish, human blood was detected in all but 2 of the 24 pools. Human sequences also dominated non-human sequences in abundance by at least an order of magnitude in most pools (Tables 2 and 3). Nine distinct human haplotypes were identified, and these differed from one another by 8 common single-nucleotide polymorphisms (SNPs) catalogued at https://www.mitomap.org (S1A Fig) [37]. The observed SNP diversity was compared with that obtained when subsampling sequences from the 1000 Genomes Project (1KGP) [38] to help gauge how many humans were fed upon by the 108 triatomines from which bloodmeals were pooled. Randomly subsampling 1KGP sequences available for Lima (Peru), Medellín (Colombia), and Puerto Rico (USA), an average subsample size of at least 75 individuals was required to observe 8 SNPs within the cytb locus amplified herein (S1B Fig). If SNP diversity in these populations is consistent with that in Caracas, results suggest that more than 2 human bloodmeals occurred for every 3 triatomines in the barcoded sample set.

Given that human cytb DNA was detected in 22 of 24 pools, no relationship between *T*. *cruzi* infection status and human vs. non-human blood feeding events was observed. Numbers of non-human bloodmeal detections were also similar between *T*. *cruzi*-positive (11 of 12) and *T*. *cruzi*-negative (11 of 12) pools. Infection status was distinct, however, in pools involving the 5 avian species detected in this study. These 4 pools were exclusively negative for *T*. *cruzi* infection.

Eleven of 14 non-human bloodmeal sources corresponded to the 2013–2016 collection period, and 9 of 14 non-human bloodmeal sources corresponded to male triatomines.

Non-human bloodmeal sources were most diverse in Baruta (8 different sources), followed by Sucre (5) and Petare parish (1).

### Reports of triatomine biting

During the 2014–2019 study time period, contributors were also asked whether or not they noticed having been bitten near the time of triatomine discovery. A total of 1153 triatomine submissions included responses to this question and were additionally assayed for the presence/absence of liquid content within the intestines (potential bloodmeal). Of these, 721 were submitted by contributors reporting bites. These bites were verified as triatomine-like by medical staff, and 85.4% (616/721) of corresponding triatomine specimens were positive for the presence of liquid content within the intestines. Forty-five of these bite- and liquid content-positive specimens also featured in the above bloodmeal metabarcoding analysis, and all belonged to pools positive for human cytb DNA. Of the 337 submissions by contributors reporting that no triatomine bite had occurred, 27.3% (92/337) were positive for the presence of liquid content within the intestines (S1 File). A chi-squared test indicates that these contributor responses on biting events predict triatomine feeding status (presence or absence of liquid content within the intestines) (p < 0.001).

Additionally, 95 specimens were associated with contributors that reported not knowing if they had been bitten or not. Of these, 61.1% (58/95) were positive for the presence of liquid content within the intestines.

## Discussion

This study assessed spatiotemporal triatomine prevalence, *T*. *cruzi* infection, and blood feeding patterns in the capital of Venezuela by analyzing > 4000 triatomine specimens encountered in domiciliary areas by inhabitants of the city and submitted to the IMT between 2007 and 2019.

The IMT received an average of 375 specimens per year during the 13-year study period, with collection highs generally occurring between April and July. The vast majority (99.01%) of these specimens were *P*. *geniculatus* and most were positive for *T*. *cruzi* infection (75.2%), with similar rates observed between sexes. Infection rates appear to increase between 2007 and 2011, then remain relatively constant until 2019. A majority (52.5%) of adult specimens, more often females (65%) than males (34.5%), also presented intestinal content suggestive of vertebrate blood. This visually blooded condition was especially likely when contributors reported having been bitten near the time of triatomine discovery. Human cytb DNA was detected in 22 of 24 visually blooded dissection pools subjected to barcoding analysis. Polymorphism levels among the detected haplotypes suggest that more than two human bloodmeals occurred for every three triatomines in the barcoded sample set. The DNA of 14 other animal species was also detected, but only in a single dissection pool each. The study additionally documented triatomine nymphs (stages II–V) in domiciliary areas in 18 of the MDC’s 32 parishes. Nymphs accounted for >5% collected specimens in various parishes with strong total triatomine collection yields (e.g., Sucre and Filas de Mariches). Nymphs were also generally positive for *T*. *cruzi* infection (58.2%) and frequently in a visually blooded state (80.9%).

This study’s recurrent observation of *P*. *geniculatus* in domiciliary areas adds to accumulating evidence for domiciliary habitat use by this primarily sylvatic species [9]. Domiciliary *P*. *geniculatus* occurrence was noted as early as 1986 in the MDC [43] and in at least seven other Chagas disease-endemic countries in later years [9,14,44–51]. Intrusive domiciliary behavior by *P*. *geniculatus* is facilitated by adult flight ranges of at least 2 km and is generally associated with nocturnal attraction to light. Climatic influences on intrusion behavior have been suggested in some triatomine species but are not well resolved for *P*. *geniculatus* [52–56]. This study’s highest collection yields generally occurred during months presenting higher rainfall in northern Venezuela (April–July), possibly reflecting climatic influences on triatomine proliferation and dispersal behavior as shown for many others insect species [57,58]. Future studies using matched data on sylvatic and domiciliary prevalence patterns may help determine whether collection peaks reflect overall abundance phenology or are specific to domiciliary intrusion events.

Numerous encounters of triatomine nymphs inside houses, now recorded in a total of 18 MDC parishes (previously 15 parishes for the period 2007–2013) [21], represents one of the key findings of this study. Given their lack of wings, nymphs are very likely to have hatched in near proximity to where they are discovered (in this study often in bedrooms and specifically beneath mattresses). Similar is true for the location of nymphal blood feeding and parasite infection. The presence of blood-fed nymphs in domiciliary areas thus strongly indicates successful courses of domiciliary reproduction and the possibility that long-term domiciliation is developing or established. The occurrence of *P*. *geniculatus* eggs and nymphal stages within domiciliary areas has been documented in various countries [14,59–61]. Some laboratory and field studies also suggest that adaptive processes are occurring in different microhabitats within the MDC [62–64]. Aldana et al., for example, suggest that intra-domiciliary specimens collected in 2008–2009 from Altagracia and Petare parishes exhibit adaptive wing size reductions relative to rural and sylvatic controls from Lara state [63]. It will be important to further quantify rates of successful reproduction and identify microniches where eggs are deposited in domiciliary areas of the MDC. Long-term triatomine domiciliation is pivotal epidemiologically because it suggests that domiciliary conditions and resources suffice for survival (food, shelter from predators, etc.) and reproduction without necessary connectivity to sylvatic ecotopes. This scenario can discouple domiciliary and sylvatic transmission cycles and implies different intervention priorities, particularly when considering the significant presence of synanthropic *T*. *cruzi* reservoirs like opossums and rats [65–67] that facilitate an urban enzootic cycle of the parasite. Measures to reduce urban habitat suitability, e.g., eliminating indoor breeding niches or limiting the availability of synanthropic feeding sources such as rodents in urban areas, may show greater success in mitigating disease risk than measures focusing on inhibiting intrusion from peripheral sylvatic environment.

Domiciliary habitat intrusion and potentially also long-term domiciliation processes become more concerning in conjunction with the high rates of *T*. *cruzi* infection this study reports from domiciliary collection. Triatomine infection rates of similar magnitude are sporadically reported in more rural or exclusively sylvatic areas [9,68,69] but very rarely in such a highly urban context. The city of Cochabamba in Bolivia is a rare example of comparable urban scale with similar levels of triatomine infection in some districts [50]. Cochabamba also suffers from high human seropositivity for *T*. *cruzi* [70]. Rates of human infection in Caracas, however, are currently unknown.

This study’s bloodmeal analysis offers insights on the transmission context underlying high rates of *T*. *cruzi* infection in domiciliary triatomines of the MDC. For example, results illuminate possible avenues of connectivity between sylvatic and urban *T*. *cruzi* transmission cycles, exposing bloodmeals on didelphid opossum and armadillo (*D*. *novemcinctus*) in samples from neighborhoods on the perimeter of the city (e.g., Barrio El Progreso). These two host mammals are strongly associated with *T*. *cruzi* lineages TcI and TcIII (respectively) in Venezuela [65,71]. Triatomine contact to such canonical reservoir hosts, perhaps primarily in semi-forested urban areas characteristic of Baruta parish, combined with large rodent reservoir populations in much of the city (especially impoverished regions such as Petare) likely contribute to the high rates of *T*. *cruzi* infection documented herein. Previous evidence from the MDC that *P*. *geniculatus* infection by *T*. *cruzi* primarily involves TcI (e.g., 269 of 270 previously analyzed infections presenting TcI and 1 of 270 presenting TcIII [21]), with TcI also often found in rats [65], further aligns with the bloodmeal barcoding results of this study. Importantly, however, bloodmeals on human hosts were detected far more often than those on common synanthropic animals such as mice/rats (identified in three pools), pigeons (one pool), or dogs (not detected). While very prominent, multiple observation of human blood feeding is not unexpected; similar has been reported from various less urban environments where human density relative to that of other animals is lower than in the MDC [69,72,73]. This study’s bloodmeal analysis also specifically addressed domiciliary triatomines found in a visually blooded state. Human blood detection occurred most often in pools of female triatomines of the sample set, with these accounting for only five of 14 non-human bloodmeal sources identified in cytb analysis (despite accounting for 68 of 108 analyzed specimens). This trend could potentially indicate sex-specific behavioral differences, for example, that female triatomines are more likely to focus activity on indoor areas that favor egg deposition and species survival as opposed to moving back and forth between indoor and outdoor areas.

Further investigation with larger and more systematically obtained intra- and extra-domiciliary sample sets is certainly required to determine the extent to which patterns observed in bloodmeal analysis reflect vector behavior and/or ecological circumstances (prey abundance, accessibility, etc.) as opposed to various methodological details–e.g., this study’s sampling focus on visually blooded specimens, and its single-marker metabarcoding approach: although the cytb fragment sequenced at high depth in this study (median of >260000 read-pairs per sample pool) represents one of the best validated markers of arthropod bloodmeal diversity [74], a multi-marker method involving also shorter DNA targets may have enhanced amplification from degraded (digested) DNA fragments and could have provided additional control against stochastic dropout or amplification bias that may affect some taxa. For example, while observed bloodmeal diversity (15 species) and hit depths are generally similar to those reported in other studies of triatomine feeding behaviour [27], the absence of dog DNA in this study’s sample set was unexpected (dogs represent a common triatomine feeding source in various contexts [27,75,76]) and could have been further verified with additional marker screening. Inferences from bloodmeal analysis were also limited by the decision to pool sets of intestinal samples before indexing. This minimized the extent to which identified bloodmeals could be tied to individual specimens. For example, while human haplotype diversity analysis helped estimate that ≥75 distinct human sources contributed to the human DNA signal detected across 108 triatomine specimens, it was not possible to delineate how these sources were distributed across individual specimens, e.g., to determine how often individual specimens fed on more than a single human individual. One reason why resolving such details is valuable relates to the probability of *P*. *geniculatus* defecation during host contact, which depends on whether feeding to repletion generally occurs via one complete bloodmeal on a single host or via multiple incomplete bloodmeals across multiple hosts [19]. A controlled experiment evaluating blood source DNA detection half-life would further improve inferences on individual triatomine bloodmeal diversity across multiple feeds and potentially help characterize individual triatomine movements between habitats associated with different types of prey. Such higher-resolution analysis might also help better understand whether the occurrence of *T*. *cruzi*-refractory animals (e.g., this study detected bloodmeals from 5 avian species as well as one species of gecko) has a significant effect on the stability of enzootic *T*. *cruzi* transmission.

We note various further complexities surrounding the citizen science-based sample collection approach. Spatial patterns in triatomine collection yield, for example, may be influenced by variable awareness of the IMT collection program and more generally of Chagas disease. Similar citizen science-based work in Texas (USA) has shown that triatomine submissions vary temporally in response to media attention [77], and this effect may have contributed to occasional aberrations in collection yield in this study (e.g., exceptional collection yield in May 2010 coincides with an oral Chagas disease outbreak among children in Caracas [78]). The probability of triatomine encounter and collection likely also differs by species and circumstances such as whether a human biting event previously occurred. Aside from representative sampling, data completion and precision levels are challenging to maintain in citizen science. As yet, only a subset of this study’s sample set could be assigned specific geographic coordinates due to difficulties in describing addresses, especially from areas where standard naming and numbering systems do not apply. Stigma likely also plays a role in the report of microgeographic details such as the precise domiciliary space (e.g., type of room or extent of insulation from the outside) in which a specimen is found or personal information such as the possible occurrence of triatomine biting events.

Taken together, this study from the capital of Venezuela shows widespread domiciliary habitat use (including cases of reproduction) by *P*. *geniculatus* triatomines exhibiting high rates of *T*. *cruzi* infection. Many specimens are encountered in a blood-fed state which participant surveys as well as molecular analyses suggest often to correspond to a recent human blood feeding event. But how do these observations translate to human risk of active vectorial infection? Unlike highly effective vectors such as adult *R*. *prolixus* and *T*. *infestans* which generally defecate within ca. 30 minutes of initiating a bloodmeal [79,80], defecation is suggested to occur within approximately one hour after bloodmeal initiation for adult *P*. *geniculatus* [19]. This interval is only slightly shorter than the average time needed to complete a full bloodmeal without interruption [19]. The likelihood that *P*. *geniculatus* feces containing infective parasites are released during human contact is therefore expected to be lower than in several other triatomine species. Vectorial capacity and human infection risk are however highly context-specific, conditioned by a multitude of parameters such as host/vector densities, infection rates, and infection intensities whose complex interplay in the unique urban epidemiological setting represented by the MDC must be further investigated. A systematic human serological survey for *T*. *cruzi* occurrence in the MDC is important to this end. Other important investigative and precautionary measures include developing a better understanding of domiciliary niches where triatomine reproduction occurs, reducing urban *T*. *cruzi* reservoir populations, promoting housing materials that reduce triatomine intrusion, tracking and intervening against patterns of parasite-food source contamination [81], further educating the public on Chagas disease epidemiology, and increasing cognizance among medical workers towards the possibility of acute *T*. *cruzi* infection and associated clinical profiles. All of these objectives are especially challenging in Venezuela’s current socioeconomic situation, and broad support in funding and resources is key.

## Supporting information

S1 File Dataset of the 4872 triatomines used in this study. This dataset contains triatomine ID code, species, state, parish, sex, age, parasitology result, and presence of liquid material (potential bloodmeal) in the intestinal content. The file features interactive dashboard elements (users may subset collection statistics by selecting categories of interest).
(XLSX)

S2 File FASTA file for of operational taxonomic units resolved in cytb analysis. Sequences include primer-binding regions.
(TXT)

S1 Table Metadata on 108 *P*. *geniculatus* specimens used to identify blood feeding sources for the MDC collection period 2009–2016. Specimens represent a random visually blooded subset from Petare, Baruta, and Sucre parishes. Nymphal stages were not analyzed.
(XLSX)

S2 Table Rates of natural *T*. *cruzi* infection in triatomine specimens for the MDC collection period 2007–2019, tabulated by species. Abbreviations ‘NEG’ and ‘POS’ denote negative vs. positive *T*. *cruzi* infection status inferred via microscopy.
(XLSX)

S3 Table Rates of natural *T*. *cruzi* infection in triatomine specimens for the MDC collection period 2007–2019, tabulated by parish. Infection status was inferred via microscopy.
(XLSX)

S4 Table Rates of observing liquid material (potential bloodmeal) in triatomine intestinal content for the MDC collection period 2007–2019, tabulated by species. Abbreviations ‘B’ and ‘NB’ denote presence and absence of blood-like intestinal content, respectively.
(XLSX)

S5 Table Rates of observing liquid material (potential bloodmeal) in triatomine intestinal content for the MDC collection period 2007–2019, tabulated by parish. The presence of blood-like intestinal content is also referred to as the ‘visually blooded’ condition elsewhere in this work.
(XLSX)

S6 Table Prevalence of nymphal triatomine stages for the MDC collection period 2007–2019, tabulated by parish. Values represent percentages of collected specimens representing nymphs.
(XLSX)

S1 Fig **A) Human cytb target diversity in pooled *P*. *geniculatus* bloodmeals.** The sequence positions and nucleotide changes distinguishing the 9 human haplotypes (green circles) detected in this study are shown in white boxes. Labels outside boxes indicate the number of times each mutation has been recorded at https://www.mitomap.org [37] to help verify sequence denoising performed using the R package DADA2 [36]. **B) Relationship between human sample size and number of segregating sites in cytb target alignment using publicly available datasets from the 1000 Genomes Project.** One hundred random cytb target segments (excluding primer binding sites) were drawn for every possible sample size from multi-fasta files representing American mitogenomes from Lima, Peru; Medellín, Colombia; and Puerto Rico (all municipalities) [38]. Segregating sites were extracted after each draw using SNP-sites v2.1.3 [41].
(TIF)
