# Correction: Vector mapping and bloodmeal metabarcoding demonstrate risk of urban Chagas disease transmission in Caracas, Venezuela

**DOI:** 10.1371/journal.pntd.0011489

**Published:** 2023-07-14

**Authors:** Maikell Segovia, Philipp Schwabl, Salem Sueto, Candy Cherine Nakad, Juan Carlos Londoño, Marlenes Rodriguez, Manuel Paiva, Martin Stephen Llewellyn, Hernán José Carrasco

The Funding statement is incomplete. The complete statement is:

This work was supported by the Fondo Nacional de Ciencia, Tecnología e Innovación (FONACIT-Venezuela) (grant G-2005000827 to HJC), the Scottish Funding Council (Small Grants Fund SFC/AN/12/2017 to MSL) and grant #204820/Z/16/Z to PS by the Wellcome Trust Institutional Strategic Support Fund (https://wellcome.ac.uk/what-we-do/our-work/institutional-strategic-support-fund). The funders had no role in study design, data collection and analysis, decision to publish, or preparation of the manuscript.
