# Correction: Assessing insecticide susceptibility, diagnostic dose and time for the sand fly *Phlebotomus argentipes*, the vector of visceral leishmaniasis in India, using the CDC bottle bioassay

**DOI:** 10.1371/journal.pntd.0011480

**Published:** 2023-07-19

**Authors:** Rahul Chaubey, Ashish Shukla, Anurag Kumar Kushwaha, Puja Tiwary, Shakti Kumar Singh, Shawna Hennings, Om Prakash Singh, Phillip Lawyer, Edgar Rowton, Christine A. Petersen, Scott A. Bernhardt, Shyam Sundar

The seventh author’s name is spelled incorrectly. The correct name is: Om Prakash Singh.
