# Supplementary figures and images for: Vector mapping and bloodmeal metabarcoding demonstrate risk of urban Chagas disease transmission in Caracas, Venezuela
Source: PLoS Negl Trop Dis. 2023 Mar 17;17(3):e0010613. doi: 10.1371/journal.pntd.0010613 (PMC10057784; doi:10.1371/journal.pntd.0010613)

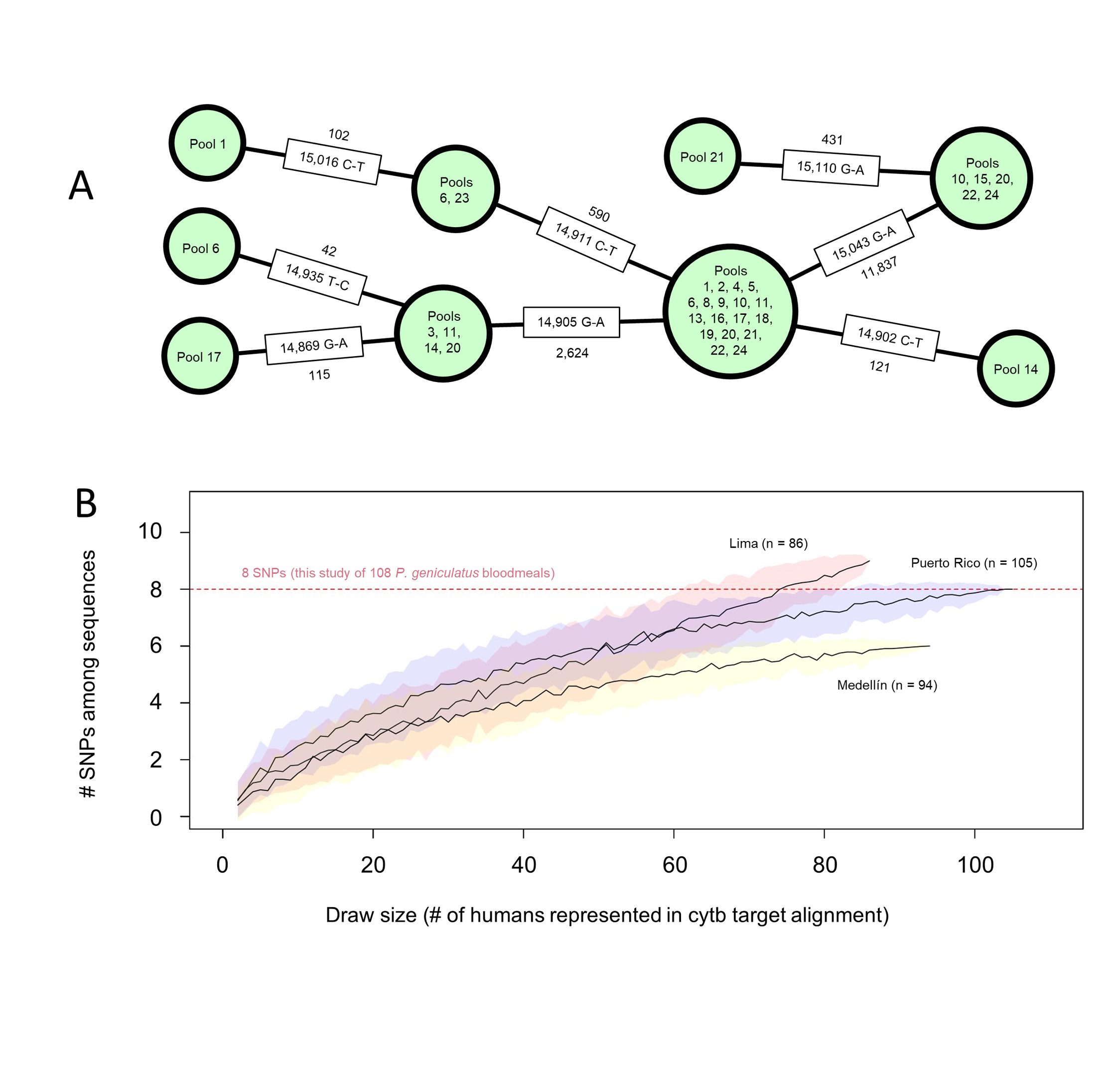

Supplement: S1 Fig — A) Human cytb target diversity in pooled P. geniculatus bloodmeals. The sequence positions and nucleotide changes distinguishing the 9 human haplotypes (green circles) detected in this study are shown in white boxes. Labels outside boxes indicate the number of times each mutation has been recorded at https://www.mitomap.org [37] to help verify sequence denoising performed using the R package DADA2 [36]. B) Relationship between human sample size and number of segregating sites in cytb target alignment using publicly available datasets from the 1000 Genomes Project. One hundred random cytb target segments (excluding primer binding sites) were drawn for every possible sample size from multi-fasta files representing American mitogenomes from Lima, Peru; Medellín, Colombia; and Puerto Rico (all municipalities) [38]. Segregating sites were extracted after each draw using SNP-sites v2.1.3 [41]. (TIF) [file pntd.0010613.s009.tif]
